# Dissociable Influences of Skewness and Valence on Economic Choice and Neural Activity

**DOI:** 10.1371/journal.pone.0083454

**Published:** 2013-12-20

**Authors:** Nicholas D. Wright, Mkael Symmonds, Laurel S. Morris, Raymond J. Dolan

**Affiliations:** 1 Wellcome Trust Centre for Neuroimaging, Institute of Neurology, University College, London, United Kingdom; 2 Nuffield Department of Clinical Neurosciences, University of Oxford, Oxford, United Kingdom; Max Planck Institute for Human Cognitive and Brain Sciences, Germany

## Abstract

Asymmetry in distributions of potential outcomes (i.e. skewness), and whether those potential outcomes reflect gains or losses (i.e. their valence), both exert a powerful influence on value-based choice. How valence affects the impact of skewness on choice is unknown. Here by orthogonally manipulating the skewness and valence of economic stimuli we show that both have an influence on choice. We show that the influence of skewness on choice is independent of valence, both across and within subjects. fMRI data revealed skew-related activity in bilateral anterior insula and dorsomedial prefrontal cortex, which shows no interaction with valence. Further, the expression of skew-related activity depends on an individual’s preference for skewness, and this was again independent of valence-related preference. Our findings highlight the importance of skewness in choice and show that its influence, both behaviourally and neurally, is distinct from an influence of valence.

## Introduction

Value-based decision-making is influenced by risk in potential outcomes, and also by whether those potential outcomes reflect gains or losses (i.e. their valence). Recent evidence suggests that risk and valence exert independent influences on choice [1]. However, in those paradigms risk was defined as the variance in potential outcomes, ignoring that a crucial aspect is asymmetry in probabilities of possible outcomes. Such skewness powerfully influences choice in foraging animals [2] and in humans performing laboratory based economic tasks [3]. Indeed, attitudes to negative skew (i.e. a small chance of a particularly bad outcome) are important in performance measurement of financial investment [4], and attitudes to positive skew (i.e. a small chance of a particularly good outcome) help explain gambling [5]. How the impact of skewness on choice is affected by outcome valence is unknown, and here we examine this relationship behaviourally and neurally.

A behavioural influence of skewness has been highlighted in recent work using options involving only gain amounts [3], [6]. Neurally, in both studies, and also when passively viewing skewed mixed gambles containing both gains and losses [7], skewness in value-based stimuli was reflected in activity within anterior insula, a region implicated in processing of uncertainty [8]–[10]. In contrast, there is a dearth of behavioural data on the relationship between skewness and valence, although the attractiveness ratings of a skewed gamble with gains can be markedly reduced by adding a loss outcome [11]. As far as we are aware studies of neural encoding of skewness have relied on either gains or mixed gambles [3], [6], [7].

We orthogonally manipulated skewness, variance and valence of value-based stimuli during economic choice. Expected Value (EV) was kept constant within these choices. Behaviourally, we tested whether skewness influenced choice, and also asked how this influence of skewness may relate to the influences of both variance and valence. Using fMRI, we asked how the influence of skewness was reflected neurally, and how this may differ according to valence, with a particular focus on anterior insula.

## Methods

### Participants and ethics statement

All participants provided written, informed consent. The University College London (UCL) Ethics Committee approved the study. All participants were healthy and were recruited using institutional mailing lists. 27 right-handed participants took part (age mean 24 years, range 18–33; 13 male; one further participant excluded who used a fixed strategy of choosing the sure option). No participants had taken part our previous studies with related paradigms [1], [3].

### Task

**Figure 1:**
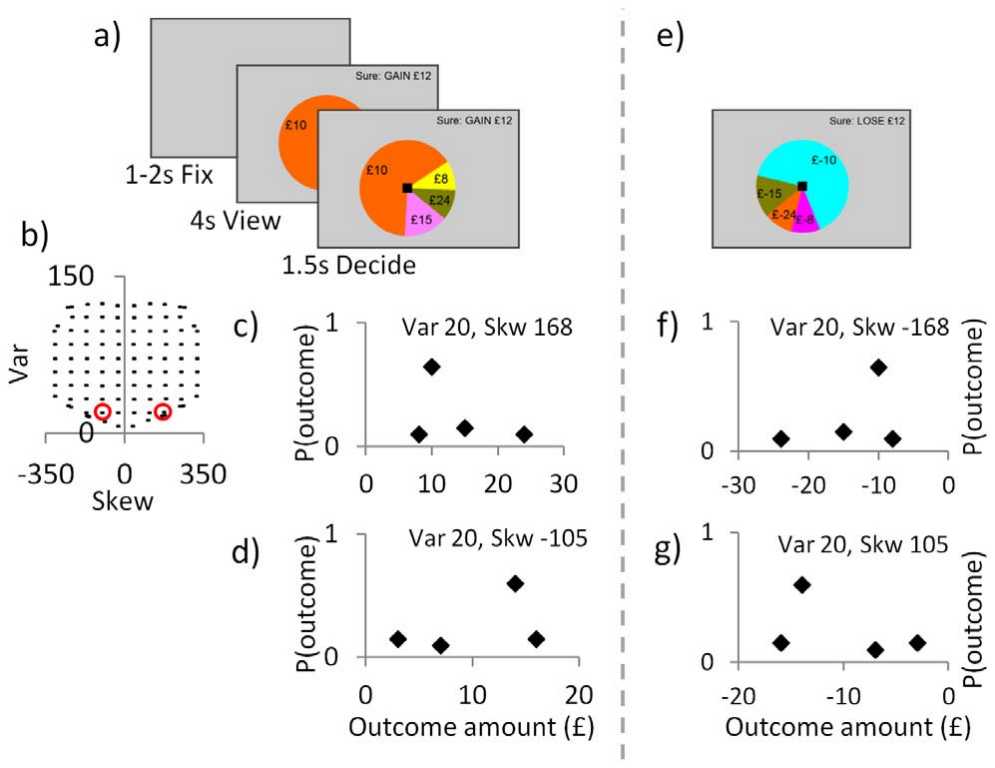
Experimental design. Our design orthogonally manipulates variance, skewness and valence in a modification of our “accept/reject” task (previously in this task we parametrically manipulated EV and lotteries were unskewed). **a**) In each “gain trial” individuals chose to accept a lottery (4 possible outcomes, all ≥0; EV £12) or reject and so receive £12 for certain. **b**) We created a set of 100 “gain trials” that parametrically and orthogonally manipulated the degree of skewness (10 levels) and variance (10 levels) of the lotteries. In this set of gain trials half had positive skew (e.g. panel **c**), and half had negative skew (e.g. panel **d**) (the two example lotteries in panels c and d are highlighted in panel b by circles). **e**) Multiplying all “gain trial” amounts by –1 gave 100 “loss trials” with identical parametric manipulations. Panel **f** shows the example lottery in panel c when it is a loss trial, and panel **g** shows the example lottery in panel d when it is a loss trial. All 200 trials were presented in random order.

In this “accept/reject” task (Fig. 1) there were 200 trials presented in a random order, of which 100 were “gain trials” (all possible outcomes ≥0) and 100 were “loss trials” (all outcomes ≤0). In each trial participants chose to accept or reject a lottery (four possible outcomes) compared to a sure option (£12 in “gain trials”; £-12 in loss trials). Each trial began with a fixation cross presented for 1-2secs (mean 1.5secs), followed by viewing the options for 4020msec; and finally a black square appeared to indicate participants had 1500msec to input their choice by button press (the black square turned white when they chose). If participants did not respond, they received £0 on a “gain trial” and the maximum loss possible on a “loss trial” (£-24).

Our decision-variables of interest were skewness, variance and valence. We controlled for the Expected Value (EV) of the lottery, which was always equal to the sure option (i.e. £12 or £-12). We manipulated the two aspects of risk by using a set of 100 lotteries (four possible outcomes, all ≥0; Fig. 1b) in which we parametrically and orthogonally manipulated the degree of skewness (10 levels, with half positively skewed and half negatively skewed) and variance (10 levels). We presented each lottery in this set once to give 100 “gain trials”. To manipulate valence, we multiplied all amounts by –1 to give 100 “loss trials” (i.e. all outcomes ≤0, and a sure option of £-12). This created a set of “gain trials” and a set of “loss trials” that were matched in their parametric modulations of skewness and variance.

Participants began the day with an endowment of £24. After the experiment, one “gain trial” and one “loss trial” were picked at random and their outcomes were added to the endowment to determine payment. Participants could receive between £0–48.

### Stimulus sets

We generated the set of 100 “gain trials” in two stages. First, we generated a list of every possible trial within the following constraints: lottery EV was £12 (i.e. equal to the sure option); each lottery had four outcomes (i.e. four pie chart segments); outcomes were between £0–£24; the smallest allowable probability was 0.1; and the smallest allowable probability increment was 0.05. Second, from within this very large number of potential trials, we selected our set of 100 trials that were the closest match to our desired 10 levels of skewness and 10 levels variance. Variance ranged from 7 to 124, skewness ranged from –317 to 317 (absolute skewness ranged from 30 to 317).

**Calculation of EV, Variance and Skewness.** For a given lottery with N potential outcomes *(m_1_, m_2_,… m_N_)*, with probabilities *p* = *p_1_, p_2_, …p_N_*, we define the EV, variance (Var) and skewness (Skw) of the outcome distribution as follows:
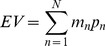
(1)

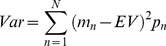
(2)

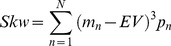
(3)


### Statistical analysis

In our behavioural analyses, statistical tests were carried out using paired or independent-samples t-tests, or mixed analyses of variance (ANOVA) in SPSS; reported p-values are two-tailed.

### Reaction time analysis

We normalised each individual’s RTs by taking the natural logarithm, mean-correcting and dividing by the standard deviation. However, we note that our findings were the same irrespective of having used “raw” or normalised RTs.

### Behavioural modelling

We modelled choice using utility functions described previously [1]. Additionally we assessed utility functions explicitly including an influence of skewness on choice. We fit data on an individual participant basis, modelling behaviour by estimating model parameters using maximum likelihood analysis implemented in Matlab. We compared models with different utility functions using Group Bayes Factors, where the Bayesian Information Criterion (BIC) penalisies model complexity [12]. In all our models, on each trial the subjective values, or utilities (*U*), of both options were computed using one of the utility functions below. These values were then compared to generate a trial-by-trial probability of each choice, using a softmax function with a free parameter *β* (constrained between 0 and 20) that allows for noise in action selection.

The behavioural modelling enables us to confirm that our manipulations of skew, variance and valence consistently influenced choice. Therefore, to illustrate the influences of variance and valence on choice we used the same two models we used previously to illustrate these effects with unskewed lotteries [1]. First, to test for an influence of variance we used a Mean-Variance model (U  =  EV + Var*ρ), in which *ρ* is a free parameter (a risk-neutral individual has *ρ* = 0, risk-averse *ρ*<0, and risk-seeking *ρ*>0). Second, to test an additional influence of valence we used the winning Mean-Variance-Valence (MVV) model from our previous datasets with unskewed lotteries [1], with a *ρ_gain_* parameter reflecting risk preference in gain trials and a *ρ_loss_* parameter reflecting risk preference in loss trials. Third, in the current study we added the influence of skewness in an MVV-skewness model (U  =  Mean + ρ*Variance + ψ*skew) with the free parameter *ψ* reflecting skew-preference in addition to those for *ρ_gain_,* and *ρ_loss_*. Note that in this study lottery EV does not contribute as it always equals the sure option. We also implemented Expected Utility, Prospect Theory and Cumulative Prospect Theory models, as previously detailed in [1].

### fMRI data acquisition

This was identical to that previously reported in [1]. Using a 3T Allegra scanner (Siemens) each participant underwent one functional run (515 volumes), acquired using a gradient-echo EPI sequence (46 transverse slices; TR, 2.76 secs; TE, 30 ms; 3×3 mm in-plane resolution; 2 mm slice thickness; 1 mm gap between adjacent slices; z-shim –0.4 mT/m; positive phase encoding direction; slice tilt –30 degrees) optimised for OFC and amygdala. We acquired a T1-weighted anatomical scan and local field maps.

### fMRI data analysis

Functional data were analysed using standard procedures in SPM8 (Statistical Parametric Mapping; www.fil.ion.ucl.ac.uk/spm). fMRI timeseries were regressed onto a composite general linear model (GLM). The GLM contained boxcars for the length of time the lottery was displayed (5.5 seconds) to examine the decision-making process. Delta functions were also included for button presses, lottery onset to account for visual stimulus presentation, and for trials in which subjects failed to respond. We modelled our neuroimaging data using a 2 valence (gain, loss) by 2 choice (risky, sure) design, as in [1]. Additional parametric modulators were included, with the height of the boxcar modulated by the skewness and variance of the lottery on each trial. Additional GLMs using modifications of this design (e.g. alternative parametric regressors) are described in the Results. The delta functions and boxcars were convolved with the canonical haemodynamic response function.

We report all activations at *P*<0.05 that survive whole brain correction using family-wise error at the cluster level [13], unless otherwise stated. Clusters were defined using a threshold of *P* <0.005. For presentation, images are displayed at *P*<0.001 uncorrected. Unless otherwise stated, small volume correction (P<0.05) was for a sphere of 8 mm radius around stated coordinates. Conjunction analyses were performed using the SPM 8 conjunction null function [14].

## Results

### Choice behaviour is influenced by skew, variance and valence

**Figure 2:**
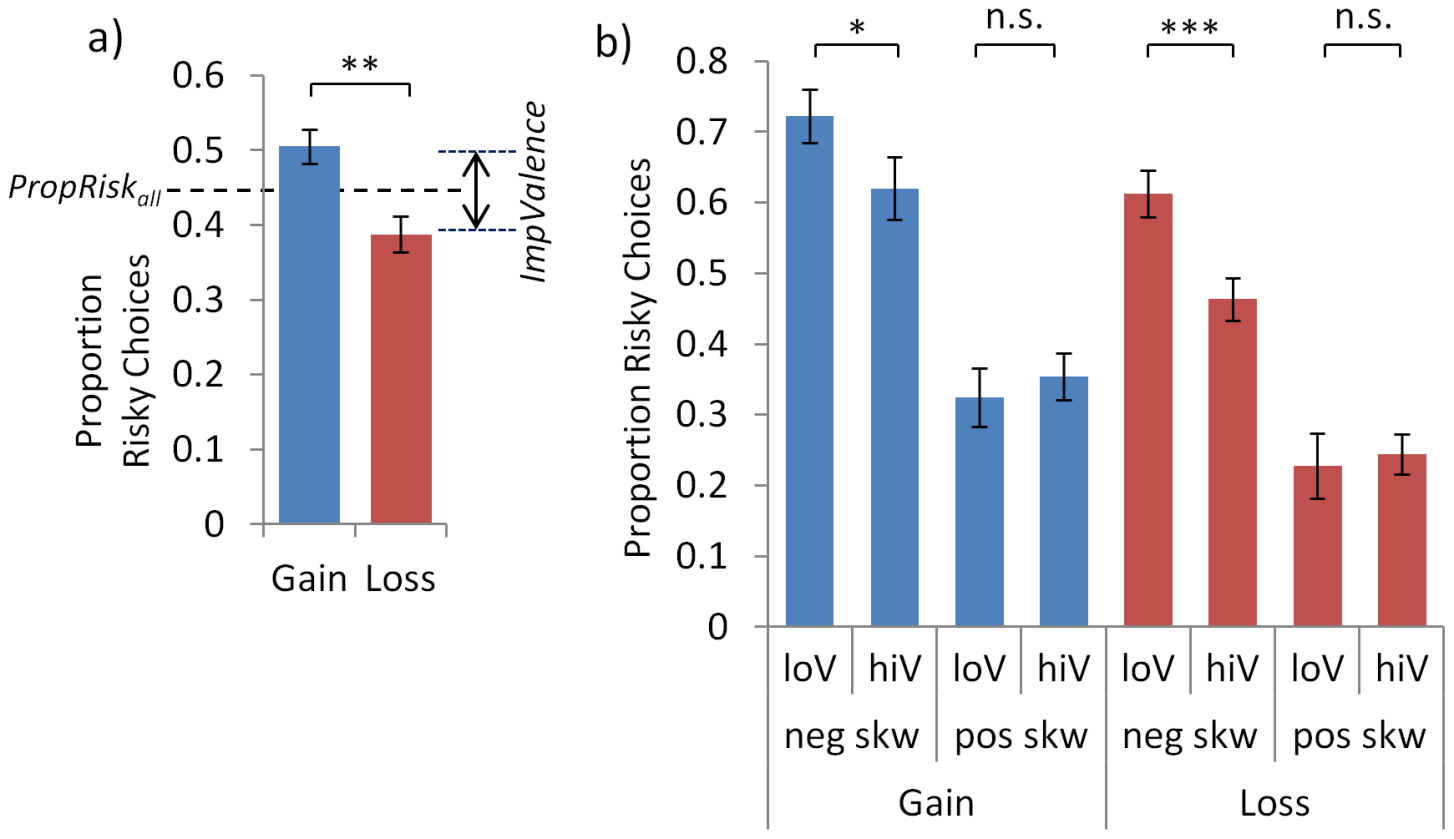
Dissociable influences of skew and valence on choice across subjects. A simple metric of risk preference as the proportion of riskier choices (PropRisk; risk-averse<0.5; risk-neutral = 0.5; risk-seeking>0.5). **a**) Individuals were risk averse overall (i.e. PropRisk_all_ <0.5). Valence also influenced choice, with more gambling for gains than losses (ImpValence  =  PropRisk_gain_-PropRisk_loss_). **b**) Choice was influenced by skew, with more gambling for negatively than positively skewed gambles. Variance only affected choice within negatively skewed gambles, such that individuals preferred low variance compared to high variance options. Error bars show s.e.m., * P = 0.04, **P = 0.001, *** P =  5×10^−5^.

The influence of risk on choice is indexed by the proportion of riskier choices made overall (*PropRisk*; risk-neutral = 0.5; risk-averse<0.5; risk-seeking>0.5). Individuals were risk-averse overall (*PropRisk_all_* 0.45±s.d. 0.09; one-sample t-test versus risk-neutral, t_(26)_ =  –3.2, P =  0.004; Fig. 2a). The impact of valence on choice is given by the difference in riskier choices in each domain (*ImpValence  =  PropRisk_gain_-PropRisk_loss_*). Individuals were sensitive to valence (*ImpValence* 0.12±s.d. 0.17; one-sample t-test versus no effect of valence, t_(26)_ = 3.6, P = 0.001), gambling more for gains (*PropRisk_gain_* 0.51±s.d.0.12) than loss outcomes (*PropRisk_loss_* 0.39±s.d.0.12; t_(26)_ = 3.6, P = 0.001). These data for risk and valence replicate previous findings in a similar paradigm, where instead we manipulated EV and variance but controlled for skew [1].

Skewness also influenced choice (Fig. 2b). Half the lotteries were positively skewed and half negatively skewed enabling us to assess the impact of skewness on choice (*ImpSkew  =  PropRisk_negSkew_ – PropRisk_posSkew_*). Individuals were sensitive to skewness (*ImpSkew* 0.32±s.d.0.20; one-sample t-test versus no effect of skew, t_(26)_ = 8.2, P< 1×10^−6^), and individuals chose the risky option more often when negatively skewed (*PropRisk*_negSkew_ 0.61±s.d. 0.12) compared to positively skewed (*PropRisk*_posSkew_ 0.29±s.d. 0.14; t_(26)_ = 8.2, P<1×10^−6^; Fig. 2b). This is the same direction of effect as in a similar paradigm manipulating skewness only with gains (Symmonds et al., 2011). Strikingly, this influence of skewness was identical regardless of whether outcomes reflected gains (*PropRisk*_GainNegSkew_ 0.67±s.d. 0.17; *PropRisk*_GainPosSkew_ 0.34±s.d. 0.16; t_(26)_ = 7.3, P<1×10^−6^); or losses (*PropRisk*_LossNegSkew_ 0.54±s.d. 0.14; *PropRisk*_LossPosSkew_ 0.23±s.d. 0.17; t_(26)_ = 7.8, P<1×10^−6^; Fig. 2b), with no interaction seen between the effect of skewness and valence (see ANOVA below).

For variance there was no simple categorical division between trial types (e.g. comparable to gain v. loss, or positive v. negative skew). However, for illustration we split trials into the half with the higher and the half with the lower variance and then assessed its impact on choice (*ImpVariance  =  PropRisk_lowVar_ – PropRisk_highVar_*). Variance influenced choice (*ImpVariance* 0.05±s.d.0.11; one-sample t-test versus no effect of variance, t_(26)_ = 2.5, P = 0.018) and subjects made more risky choices when the variance was low (*PropRisk*_LowVar_ 0.47±s.d. 0.09), than when it was high (*PropRisk*_HighVar_ 0.42±s.d. 0.11; t_(26)_ = 2.5, P = 0.018; Fig. 2b). There was no interaction with valence (see ANOVA below) but was an interaction with skewness in both gains and losses such that variance influenced choice with negatively but not positively skewed gambles (Fig. 2b).

Summarising these findings across subjects, a 2 valence (gains, losses)×2 skew (positive, negative)×2 variance (high, low) ANOVA (Fig. 2b) revealed significant main effects of skew (F_(1,26)_  = 67, P = 1×10^−8^), valence (F_(1,26)_  = 13, P = 0.001), variance (F_(1,26)_  = 6, P = 0.02) and no interaction except for that between skewness and variance described above (F_(1,26)_  = 7, P = 0.01).

### Individuals’ skew preferences were independent of those for valence or variance

**Figure 3:**
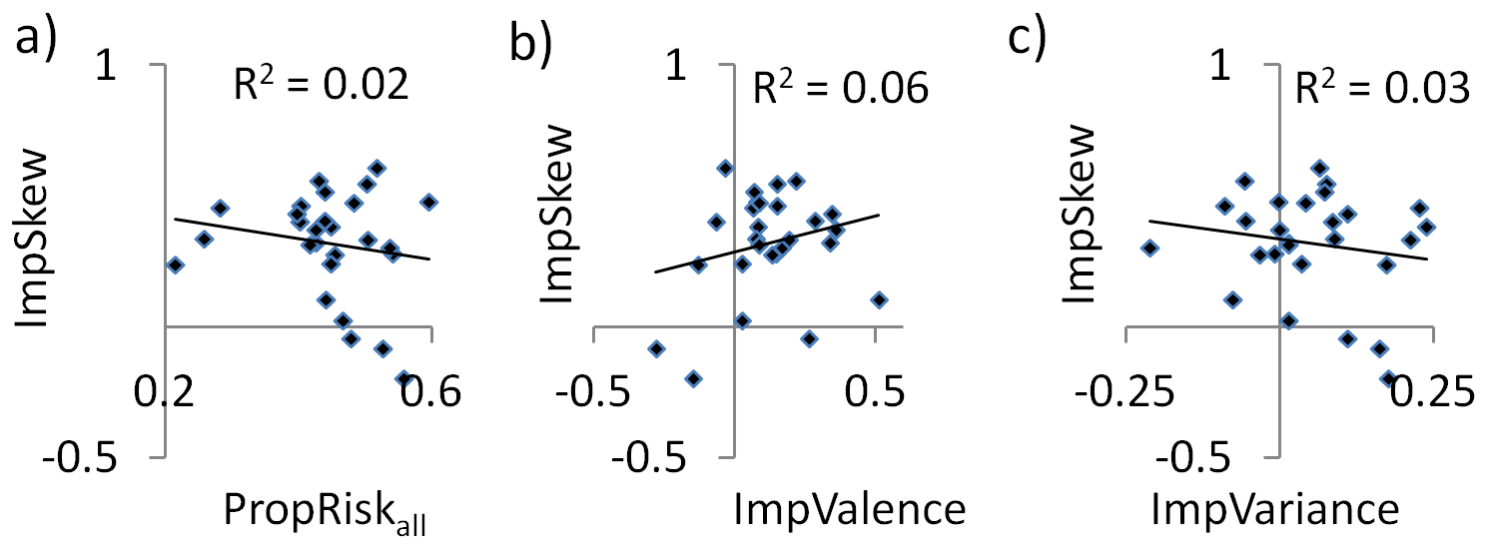
Individuals’ preferences for skew were independent of those for other influences. The impact of skew on an individual’s choices (*ImpSkew*) did not predict the effects of: **a**) risk overall (i.e., proportion of risky choices, *PropRisk_all_*); **b**) the impact of valence (*ImpValence*); **c**) or the impact of variance (*ImpVariance*) (all correlations p > 0.2).

We next examined inter-individual differences, asking if an individual’s sensitivity to one decision variable predicted their sensitivity to another. The influence of skewness on an individual’s choices (*ImpSkew*) did not predict the influence of valence (*ImpValence v ImpSkew*: r = 0.24, P = 0.2; Fig. 3) nor variance (*ImpVariance v. ImpSkew*: r = –0.17, P = 0.4) nor the proportion of risky choices they made overall (*PropRisk_all_* v. *ImpSkew:* r = –0.17, P = 0.4).

The proportion of risky choices an individual made overall (*PropRisk_all_*) did not predict the influence of valence (*PropRisk_all_ v. ImpValence:* r = –0.02, P = 0.9) nor variance (*PropRisk_all_ v. ImpVariance:* r = –0.25, P = 0.2). However, we noted a significant correlation between individuals’ preferences for variance and valence (*ImpVariance v. ImpValence*: r = –0.45, P = 0.02; i.e. more gambling for gains than losses was associated with more gambling for higher than lower variance options).

### Behavioural modelling confirmed that skew, variance and valence influenced choice

The purpose of the behavioural modelling was to confirm that our manipulations of skew, variance and valence consistently influenced choice, as shown by comparing our “summary statistic” models. To illustrate the influences of variance and valence on choice we used the same two models used previously to illustrate effects with unskewed lotteries [1]. As before, the influence of variance was captured by a Mean-Variance model that correctly predicted 59%±s.d.7% of choices (summed BIC  = 7094). The fit of the model was improved by adding valence, shown in the winning Mean-Variance-Valence (MVV) model in our previous datasets with unskewed lotteries [1] (BIC =  7003). In turn, that model was improved by also accounting for skew in our MVV-Skew model (MVVS BIC =  6102). Notably, this MVVS model also outperformed more standard Expected Utility, simple Prospect Theory and more complex Cumulative Prospect Theory models (with the reference point as zero or as a free parameter), and a modified MVVS model with separate skew parameters for positive and negative skew. In absolute terms the MVVS model correctly predicted 73%±s.d.7% of choices.

### Reaction times

**Figure 4:**
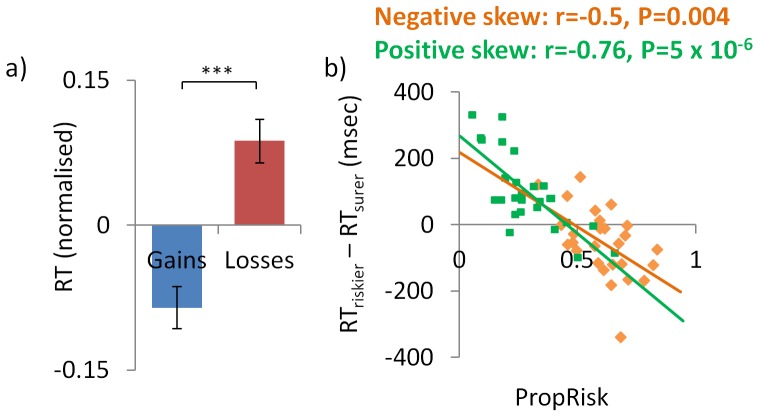
Reaction times. **a**) Individuals were slower to choose options entailing losses compared to gains. **b**) Inter-individual differences in preference for negatively or positively skewed gambles predicted their reaction time effects. Risk preference with negatively skewed gambles predicted the RT bias when approaching the riskier relative to the surer option (RT_riskier_-RT_surer_) with negatively skewed gambles; and the same was seen with positively skewed gambles. The same effects are observed when using both raw and normalised RT data (normalised shown here). Error bars indicate s.e.m.. *** P = 6×10^−4^.

Reaction times (RTs) also showed influences of skew, variance and valence (Fig. 4). Further, these data were consistent with each acting as appetitive or aversive stimulus features, where it is known that individuals are slower to approach aversive stimuli and faster to approach appetitive stimuli [15]. We have further examined and discussed such RT effects previously [1]. Regarding valence, individuals were slower to approach (choose) options entailing loss compared to gains (gains RT 594±s.d.90msec; losses 643±94; t_(26)_ =  –3.8, P = 0.001).

Regarding risk, this can be aversive, neutral or appetitive depending on an individual’s risk preference. We found that individuals’ risk preference with gains (*PropRisk_gain_*) strongly predicted RT differences when approaching (choosing) the riskier relative to the sure option (i.e. RT_riskier_-RT_surer_) with gains (r = –0.58, P = 0.001); and risk preference with losses (*PropRisk_loss_*) strongly predicted the RT difference with losses (r = –0.48, P = 0.01). Furthermore, the pattern was exactly as predicted where risk-averse individuals were slower to approach risk; risk-neutral showed no RT difference; and risk-seeking subjects were faster to approach risk.

Both aspects of risk (skewness and variance) showed the same pattern. Individuals’ risk preference with negatively skewed gambles (*PropRisk_negSkew_*) strongly predicted the RT difference when approaching (choosing) the riskier relative to the surer option (RT_riskier_-RT_surer_) with negatively skewed gambles (r = –0.52, P = 0.005); and risk preference with positively skewed gambles (*PropRisk_posSkew_*) strongly predicted the RT difference with positively skewed gambles (r = –0.74, P = 9×10^−6^; Fig. 4). Individuals’ risk preference with high variance gambles (*PropRisk_highVar_*) predicted the RT difference with high variance gambles (r = –0.58, P = 0.001); and risk preference with low variance gambles (*PropRisk_lowVar_*) strongly predicted the RT difference with positively low variance gambles (r = –0.38, P = 0.048).

### Neural data

We first tested for a representation of skewness in the stimuli. Initially, we used the same 2 valence (gain, loss) by 2 choice (sure, risky) factorial design as in our previous study with unskewed lotteries [1], but here with lottery skewness and variance as trial-by-trial parametric regressors. Activity is whole-brain cluster-level corrected (P<0.05) unless otherwise stated. We replicated the main effects of valence (greater activity for gains than losses in bilateral striatum and obitofrontal cortex (OFC); greater activity for losses than gains in pre-SMA/dmPFC and SVC in bilateral anterior insula) and choice (risky>sure in posterior parietal cortex) in the factorial analysis. Again parietal activity correlated with the parametric manipulation of lottery variance (Table S1). However, no activity correlated with skewness in that GLM, nor in an alternative GLM using the modulus of the skewness (i.e. the unsigned magnitude of the skewness in each trial).

**Figure 5:**
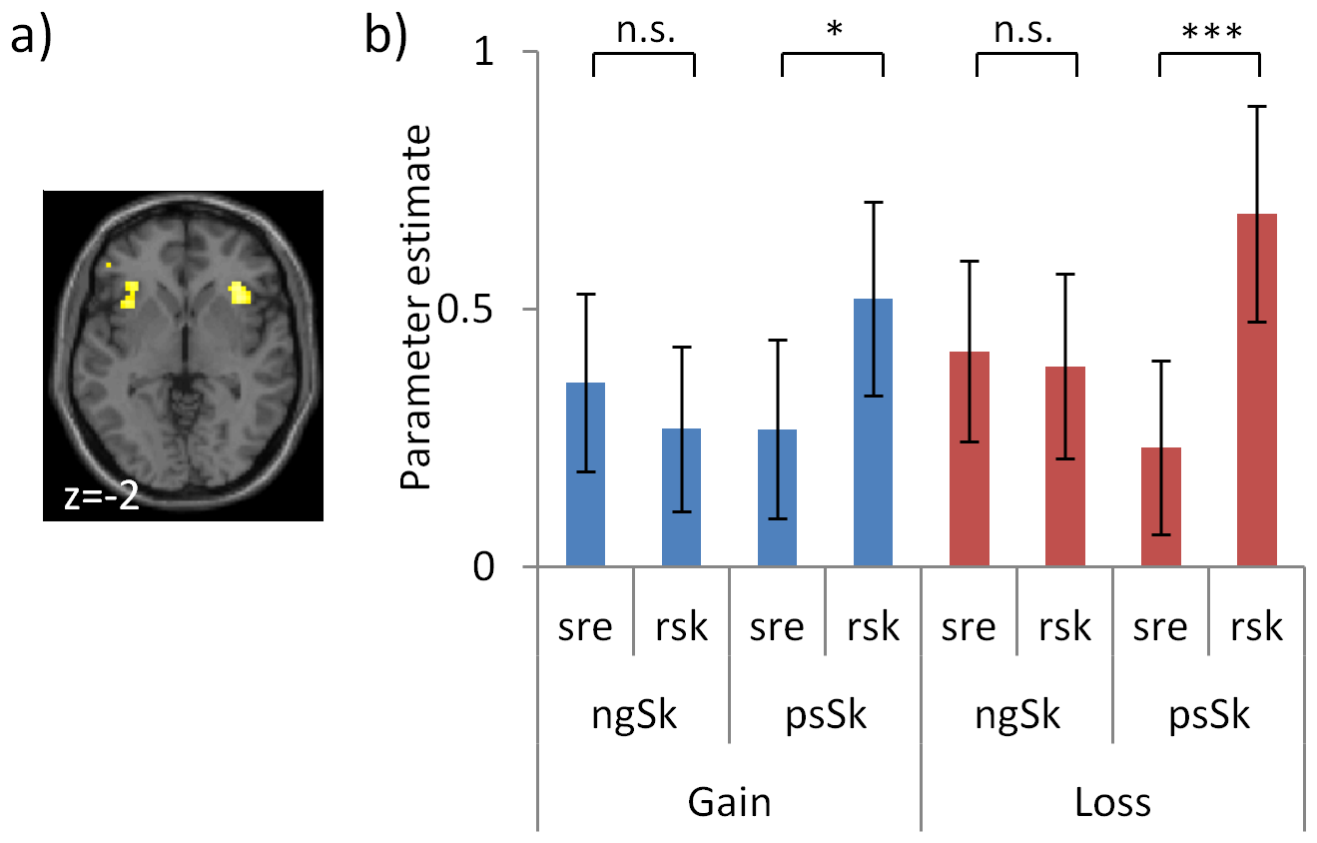
Choosing skewed lotteries alters anterior insula activity. Skewness may affect action-selection by influencing the disposition to approach economic stimuli. We examined neural activity in a GLM with a 2 valence (gain, loss) by 2 skew type (posSkew, negSkew) by 2 choice (risky, sure) factorial design corresponding to the categorical distinctions in our design, and with variance as a parametric modulator. **a)** There was an interaction of skew type by choice in bilateral anterior insula. As shown in **panel** **b** this was driven by increased activity for choosing (approaching) the risky option with positive skew, which was the specific action to which individuals were least disposed behaviourally. Further, as shown in **panel b** this pattern of skew-related activity did not interact with outcome valence. Parameter estimates plotted for the peak of the interaction in right anterior insula. Error bars indicate s.e.m.. *<0.05, **<0.005, ***<0.001

However, robust activity was seen for skewness as it interacted with choice (Fig. 5; Table 1). This was shown in a new GLM with a 2 valence (gain, loss) by 2 skew type (posSkew, negSkew) by 2 choice (risky, sure) factorial design corresponding to the categorical distinctions in our design, and with variance as a parametric modulator. An interaction of skew type by choice in bilateral anterior insula and pre-SMA/dmPFC (Fig. 5a) was driven by increased activity for choosing a risky option with positive skew (Fig. 5b), which was the specific action to which individuals were least disposed behaviourally (Fig. 2b). Further, this pattern of skew-related activity did not interact with outcome valence (Fig. 5b; no suprathreshold voxels for an interaction with valence within 10 mm of the peak in each cluster) and conjunction analysis between this activity in gains and losses revealed activity regardless of valence in these same areas (pre-SMA/dmPFC and right anterior insula). We also note that, as above, activity in this GLM corresponded to the manipulations of valence (gain>loss in OFC and striatum; and loss>gain SVC in dmPFC/pre-SMA) and variance (positive correlation in parietal cortex; Table 1).

**Table 1:** fMRI results across subjects.

Region	L/R	x	y	z	Z	#vox	p
***Gain > Loss***							
Ventral Striatum		18	5	–8	5	233	0.002
Putamen	R	21	20	–5	4.9		
		27	11	–5	4.8		
Putamen	L	–21	8	–2	4	271	8E-04
		–15	17	–2	3.9		
Amygdala		–18	–1	–17	3.9		
OFC	L	0	44	–17	4.1	177	0.011
dmPFC	R	9	68	13	3.4		
OFC		6	62	–5	3.3		
Supr. Frontal gyr.	L/R	18	41	46	4.6	322	2E-04
Supr. Medial gyr.		–3	44	49	3.7		
		–15	32	55	3.6		
***Riksier > Surer***							
Postr. Parietal gyr.	R	42	–73	37	4.7	1551	<1E-12
		27	–67	34	4.7		
Occipital		33	–79	40	4.6		
Supramarginal gyr.	L	–51	–37	31	4.2	442	<1E-04
Supr. Parietal lobule		–24	–76	46	4.1		
Supramarginal gyr.		–51	–49	34	4.1		
Mid. Cingulate/Postr.	L/R	3	–34	40	4	243	0.003
		18	–19	43	3.5		
		–9	–19	37	3.4		
IFG (p. Tri)	L	–48	35	16	4.5	495	<1E-05
Mid. Orbital gyr.		–45	50	–5	4.1		
IFG (p. Tri)		–51	35	7	4		
Mid. Frontal gyr.	R	24	11	49	4.5	1242	<1E-10
		51	38	19	4.3		
		36	29	40	4		
Precentral gyr.		–48	5	40	3.8	159	0.025
IFG (p. Oper)		–48	8	25	3.8		
Infr. Temporal gyr.	L	–48	–46	–11	4.3	137	0.048
		–57	–52	–5	3.9		
Cerebellum		–39	–55	–26	3.3		
Mid. Temporal gyr.	R	54	–49	–2	3.5	171	0.018
Fusiform gyr.		39	–46	–14	3.5		
Mid. Temporal gyr.		60	–37	–8	3.3		
***Interaction: posSkew>negSkew & riskier>surer***							
Antr. Insula	R	36	26	1	4.3	170	0.023
		36	20	–11	4		
IFG (p. Tri)		36	29	10	3.3		
Antr. Insula	L	–33	17	1	3.8	187	0.015
IFG (pars Tri)		–33	29	–2	3.6		
		–51	41	1	3.6		
ACC	L/R	–9	32	25	3.6	223	0.006
pre-SMA/SMA		0	20	52	3.4		
Mid. Cingulate cortx		6	26	37	3.3		
***Variance (pos. correl.)***							
Postcentral gyr.	R	63	–7	31	3.8	199	0.007
		63	–16	31	3.8		
Supramarginal gyr.		60	–25	40	3.6		

This table shows all activity surviving cluster level correction across the whole brain (P<0.05 FWE corrected; threshold of P<0.005 used to define the clusters) for our GLM with a 2 valence (gain, loss) by 2 skew type (posSkew, negSkew) by 2 choice (risky, sure) factorial design corresponding to the categorical distinctions in our design, and with variance as a parametric modulator. We report all activity for all main effects and contrasts in the factorial design, and for positive and negative correlations with variance and for the interaction of variance in gains versus losses. Activity for loss>gain survived SVC in dmPFC/pre-SMA (9 17 46, Z = 3.94, 103vox). For each cluster is shown: the three constituent peaks with the highest Z-scores; the number of voxels at P<0.005 (uncorrected); and the P-value of the cluster after FWE correction across the whole brain. (ACC  =  Anterior Cingulate Cortex; IFG  =  Inferior Frontal Gyrus; OFC  =  orbitofrontal cortex; SMA  =  Supplementary Motor Area).

Next we asked if inter-individual differences in sensitivity to skewness were also reflected neurally. Applying our impact of skew metric (*ImpSkew*) to the contrast that revealed skew-related activity above (i.e. the interaction of skew type and choice), demonstrated a negative correlation with activity in regions including hippocampus and OFC (Table 2). This indicated that the less susceptible an individual was to skewness (i.e. lower *ImpSkew*), the more significant the interaction of choice and skew, driven both by greater activity for risky>sure choices in posSkew trials, and lower activity for risky>sure choices in negSkew trials. This correlation was specific to *ImpSkew*, as skew-related activity did not correlate with *PropRisk_all_*, *ImpValence* or *ImpVariance*. Further whilst activity for risky>sure choices correlated with overall risk preference (i.e. *PropRisk_all_*) in anterior insula (Table 2), this was again specific and did not correlate with *ImpSkew, ImpValence* or *ImpVariance*. Finally, for completeness we note there was no correlation between variance-related activity and the *ImpVariance*, nor between activity for gains versus losses and *ImpValence*.

**Table 2:** fMRI results between subjects.

Region	L/R	x	y	z	Z	#vox	p
***ImpSkew (neg. correl.) on interaction of posSkew>negSkew and risky>sure***							
Hippocampus	L	–27	–16	–20	4.6	456	<1E-04
Supr. Temporal gyr.		–57	2	–8	3.7		
		–57	–7	–14	3.6		
OFC	L/R	3	41	–17	4.2	241	0.003
		15	53	–8	3.8		
		–3	59	–2	3.4		
Postcentral gyr.	L	–27	–28	70	3.8	203	0.008
		–15	–31	58	3.8		
Precuneus		–9	–40	58	3.5		
Precentral gyr.	R	15	–22	73	3.8	240	0.003
		36	–13	64	3.8		
		48	–7	55	3.8		
***PropRisk_all_ (neg. correl.) on risky>sure***							
Antr. Insula/IFG	L	–30	26	–11	4.3	150	0.028
		–36	17	–5	4.3		
		–39	17	10	3.8		
pre-SMA/dmPFC	L/R	6	17	49	5.3	609	<1E-06
		–6	26	46	4.9		
		–3	35	34	4.6		
Infr. Parietal lobule	L	–39	–52	37	5.1	909	<1E-08
		–45	–46	46	5.1		
Postr. Parietal ctx.		–48	–55	37	4.9		
Precuneus	R	9	–67	40	5.3	939	<1E-08
Postr. Parietal ctx.		42	–64	43	5.3		
Infr. Parietal lobule		39	–52	43	4.6		
Mid. Frontal gyr.	L	–39	17	37	4.3	337	<1E-03
IFG (p. Tri		–42	26	22	4.3		
IFG (p. Oper)		–51	20	34	3.9		
IFG (p. Oper)	R	51	5	19	4.5	1095	<1E-09
		45	17	34	4.4		
Mid. Frontal gyr.		42	38	31	4.4		
Cerebellum	R	36	–70	–44	4.4	279	<1E-03
		33	–49	–26	3.7		
		27	–40	–26	3.5		

This table shows all activity surviving cluster level correction across the whole brain (P<0.05 FWE corrected; threshold of P<0.005 used to define the clusters) in the 2 valence by 2 skew type by 2 choice model, for contrasts involving: the second level covariate for risk overall (*PropRisk_all_*) on activity for accept>reject; the second level covariate for skew (*ImpSkew*) on the skew related activity seen across subjects (interaction of skew type and choice). For each cluster is shown: the three constituent peaks with the highest Z-scores; the number of voxels at P<0.005 (uncorrected); and the P-value of the cluster after FWE correction across the whole brain.

Finally, we asked if neural activity correlated with unified subjective value (SV) derived from the winning behavioural model. In contrast to the robust findings above, No such correlation was seen (whole brain corrected or using SVC in vmPFC, OFC and striatum as regions of interest specified in the PickAtlas toolbox [16] in a GLM as above but with lottery SV as the parametric modulator, nor when using chosen minus unchosen SV, nor using the difference in SV between options.

## Discussion

Here we demonstrate that the skewness of outcome distributions influenced choice behaviour. Furthermore, this influence of skewness on choice was independent of valence both across (Fig. 2b) and between (Fig. 3a) subjects. Neurally, we observed skew-related activity across subjects in anterior insula, a region implicated in aversive representations [17], [18], and between subjects correlating with individuals’ skew preference in hippocampus and vmPFC/OFC. Mirroring our behavioural findings these patterns of skew-related activity were seen for gains and losses (Fig. 4b). These data support the idea that risk is not monolithic, either in terms of its behavioural effects or neurally, and instead that distinct aspects of risk including variance and skewness can powerfully influence choice.

The observation that skewness influenced choice behaviour is consistent with human [3], [6] and animal data [2]. Our data help characterise this influence of skewness in two further ways. Firstly, we dissociate this influence of skewness from other aspects of risk. Secondly, individuals’ preferred negative to positive skew, which replicates a recent study using a similar format [3] and previous work showing negative skew preference on average [19]. We note that other studies have shown a mixture of participants with positive or negative skew-seeking [20] or positive skew seeking behaviour [21], [22].

Neurally, we showed that skew-related activity was distinct from the activity related to other aspects of risk. Skew-related activity here was found in anterior insula, a region implicated in two previous studies of skewness in gains [3], [6] and when passively viewing skewed mixed gambles [7]. Anterior insula is a region also implicated in processing of uncertainty more generally [8]–[10]. In line with other recent work with unskewed gambles [1], this skew-related activity here was contingent on choice, and specifically there was increased activity in anterior insula when choosing the least preferred option (i.e. choosing the risky option when it contained positive skew), where anterior insula is known to be implicated in aversive representations [18].

We note that if anterior insula plays such a role we would also expect increased activity here for choosing the lottery with losses, as we have shown previously [1]. However, in this experiment skew-related preference dominated behaviour (Fig. 2b) which may have reduced sensitivity and we do not see strong evidence for such valence-related activity. Tentative evidence for this here is seen in the simpler GLM that collapsed across skewness type and that showed greater activity for losses than gains (Results above and Table S1), as well as in our main GLM where Fig. 5b shows a tendency towards a greater effect of risky than sure choices in losses than in gains. This could be usefully examined in future work.

In addition to such skew-related activity across subjects, between subjects we noted brain regions that integrate this skew-related activity with individuals’ preferences for skewness. This was seen in hippocampus, a region identified with reward in a meta-analysis of value-based choice [23] and implicated in goal-directed behaviour [24]. It was also seen in OFC, a region implicated in an integration of preference and reward related activity [25]. In these regions, the less susceptible an individual was to skewness (i.e. lower ImpSkew), the greater the interaction of choice and skewness. This closely parallels previous findings between subjects, where for example in a framing task participants who showed less framing exhibited greater OFC activity associated with the interaction of choice and frame [26]. As those authors speculate, individuals less susceptible to the frame may be better at representing their own affective influences, which enables them to modify their behaviour.

The influence of skewness on choice behaviour was independent of outcome valence both across (Fig. 2b) and between (Fig. 3a) subjects. The relationship between this aspect of risk (skewness), and valence is important as the prevailing view in psychologically-informed economics is that risk and valence have a specific relationship, in which individuals are risk-averse with gains and risk-seeking with loss outcomes [27], [28]. Contrary to this we observed that individuals chose the risky option more often with gains than with losses, which precisely replicates our previous findings in a similar paradigm with unskewed gambles [1]. Specifically regarding the relationship between the influences of skewness and valence on choice behaviour, there has not to our knowledge been a previous characterisation of how the influence of skewness (e.g. positive and negative skew) is affected by an orthogonal manipulation of valence. Supporting the idea that these may be distinct influences, however, we note that a study assaying attractiveness ratings of gambles showed such ratings were dramatically altered by adding a loss to a skewed gamble with gains (i.e. it became a mixed gamble), suggesting such a gamble may be rendered qualitatively different [11].

Skew-related activity was distinct from that related to valence (Fig. 5b). Previous studies of skewness have used either only gains or mixed gambles [3], [6], [7]. That risk and valence may exert their influence on choice independently is consistent with mounting evidence that choice is the product of multiple interacting value and decision systems, which may for example reflected in both our current and previous data [1] by distinct behavioural effects and neural substrates associated with risk and valence.

Finally, we note that our data also speak to a debate in the literature between two main competing accounts for recent neuroscientific studies examining the neural basis of risky economic choice: that “summary statistics” describe the distribution of possible outcomes from a risky choice [9], [29]; or that subjective value (SV) is determined by the shape of a utility function, with risk-preference emerging as a by-product of that shape [30]. Here consistent with a “summary statistic” account we find neural activity related to skew and variance, whilst in contrast to these robust data, there was not equally clear evidence for encoding of SV. However, we note that absence of evidence is not evidence of absence. Further, an important potential reason is provided in a recent study showing that SV representations of more complex multi-attribute stimuli are distributed, and so detectable using multivariate but not standard mass univariate analysis [31].

In conclusion, risk exerts a powerful influence on value-based decision-making and our data help parse the different dimensions of risk. The influence of skewness on choice can be dissociated from other aspects of risk both in terms choice behaviour and its neural representation. Furthermore, our data show that the influence of skewness can be dissociated from the influence of valence on choice. These data provide evidence that distinct aspects of value-based stimuli exert their influence through a choice architecture wherein multiple interacting systems contribute to choice.

## Supporting Information

Table S1 **fMRI results for 2 valence (gain, loss) by 2 choice (risky>sure) GLM.** Replicating the 2×2 analysis used in our previous study that used unskewed lotteries (Wright et al., 2012) revealed similar results across and between subjects. **Panel a) across subjects:** shows all activity surviving cluster level correction across the whole brain (P<0.05 FWE corrected; threshold of P<0.005 used to define the clusters) for our GLM with a 2 valence (gain, loss) by 2 choice (risky, sure) factorial design, with variance and skewness as parametric modulators. We report all activity for all main effects and contrasts in the factorial design, and for positive and negative correlations with the parametric modulators and for the interaction of the parametric modulators in gains versus losses. Note also activity for the contrast of loss>gain survived SVC in left AI (−36 23 1 Z = 4.01, 64vox) and right AI (36 23 7 Z = 3.78, 45vox). **Panel b) between subjects:** shows activity in this GLM for the second level covariate for risk (*PropRisk_all_*) on activity for risky>sure; and the second level covariate for valence (*ImpValence*) on activity for gain>loss. For each cluster is shown: the three constituent peaks with the highest Z-scores; the number of voxels at P<0.005 (uncorrected); and the P-value of the cluster after FWE correction across the whole brain.
(XLSX)
